# Supplementary material for: Multi-order hyperbolic graph convolution and aggregated attention for social event detection
Source: PLoS One. 2025 Dec 9;20(12):e0337540. doi: 10.1371/journal.pone.0337540 (PMC12688158; doi:10.1371/journal.pone.0337540)
Supplement: Attachment — Submitted filename: Response to Reviewer.docx [file pone.0337540.s002.docx]

**Response to Reviewer#1 Comment 1**

**Reviewer#1 Comment 1**

*The manuscript lacks a systematic comparison with existing methods to substantiate its claimed innovations. For instance, in Section 2 (“Related Work”), methods such as MOGANED and HSED are referenced, but the unique advantages of MOHGCAA—such as specific advancements in multi-order modeling or the benefits of the attention mechanism—are not clearly delineated.*

**Author Response:**

We thank the reviewer for highlighting this important issue. We agree that our original Related Work section did not clearly articulate how our method advances over existing approaches such as MOGANED and HSED.

To address this, we have revised the end of **Section 2.3** to explicitly compare MOHGCAA with prior work. Specifically:

1. **Clarified Differentiation from MOGANED and HSED**

We now explain that while MOGANED supports multi-order attention aggregation, it operates in Euclidean space and lacks the ability to model hierarchical relationships. Conversely, HSED models hyperbolic embeddings but does not incorporate multi-order dependencies or adaptive attention.

2. **Positioned MOHGCAA’s Unique Contributions**

We highlight that MOHGCAA combines both strengths by applying multi-order graph convolutions in the tangent space of hyperbolic geometry and introducing a curvature-aware attention mechanism based on Ollivier Ricci curvature.

3. **Added a Comparison Table (Table 1)**

To support this analysis, we have included a comparative table that contrasts our model against MOGANED and HSED in terms of space, order modeling, attention design, and suitability for SED tasks.

**Location of Revisions in Manuscript:**

- 1 Introduction – final paragraph (Page 3)
- Section 2.3 – final paragraph (Page 4-5)
- Table 1 – added after Section 2.3 (Page 5)

We thank the reviewer for this helpful suggestion, which allowed us to substantially improve the technical clarity and correctness of the manuscript.

**Response to Reviewer#1 – Comment 2**

**Reviewer#1 Comment 2**

*Critical technical details are inadequately explained. For example, Section 4.1.3 mentions using Ollivier Ricci curvature for attention weight computation but lacks specific formulas or derivations, hindering comprehension.*

**Author Response:**

We appreciate the reviewer’s comment and fully agree that the original formulation in Section 4.1.3 lacked sufficient clarity and mathematical rigor. We have taken the opportunity to revise this section extensively and correct a previously inaccurate reference to “Ollivier Ricci curvature,” which is **not used** in our final model. This statement has been removed to avoid confusion.

In the revised manuscript, we now provide a complete and self-contained definition of the multi-order graph convolution operation used in our model. Specifically:

1. **We clearly define the convolution function** applied to each node, incorporating order-specific neighborhood structure and learnable transformations.
2. **Attention weights are computed at the edge level**, based on a softmax normalization over unnormalized scores that are derived from a combination of node feature vectors.
3. **All mathematical formulas are provided**, including:
   - the multi-order convolution equation;
   - the softmax attention weight expression;
   - and the unnormalized score formulation using linear projections and a non-linear activation (LeakyReLU).

These updates eliminate ambiguity and allow for full reproducibility of our method. The revised section now explicitly defines all variables, operations, and learnable parameters involved in the attention-guided multi-order convolution process.

**Location of Revisions in Manuscript:**

- Section 4.1.3 – equations (6), (7), and (8); (Page 8)
- Removed all references to Ricci curvature (Page 8)

We thank the reviewer again for this helpful suggestion, which allowed us to substantially improve the technical clarity and correctness of the manuscript.

**Response to Reviewer#1 – Comment 3**

**Reviewer#1 Comment 3**

*It would be helpful to provide further insights or analysis of the performance results, especially Table 4 and Table 6. For instance, why does MOHGCAA perform significantly better on mini-Twitter or Cora? Does the nature of the dataset (e.g., number of features, hierarchy) affect the performance gains?*

**Author Response:**

We thank the reviewer for this thoughtful and constructive comment. We fully agree that a more detailed interpretation of the performance results across datasets is necessary to better understand the strengths and limitations of MOHGCAA.

To address this, we have added a new subsection titled **“5.4.3 Dataset Characteristics and Their Impact on Performance”** to the Discussion section. This subsection provides an in-depth explanation of why our model shows stronger improvements on datasets such as **mini-Twitter** and **Cora**.

Specifically:

- **mini-Twitter** contains dense interaction graphs with strong reply/retweet hierarchies and noticeable community structure. These characteristics are particularly well-suited for **hyperbolic embedding**, which captures hierarchical and scale-free patterns efficiently. Moreover, the **curvature-aware attention** mechanism in MOHGCAA dynamically adapts to such structural features, leading to substantial gains.
- In **Cora**, the citation relationships exhibit moderate depth and natural topic hierarchies. Although less explicit than in mini-Twitter, the benefit of **multi-order aggregation** is still evident, helping the model outperform Euclidean baselines. In contrast, datasets with flatter topology or weaker hierarchy (e.g., Citeseer) yield less pronounced improvement.

This analysis helps clarify that MOHGCAA’s relative performance gain is positively correlated with the **hierarchical depth, structural diversity, and semantic granularity** of the dataset. The new content enhances the interpretability of results tables, and supports our claim that the model is particularly well-suited for event-centric and socially structured data.

**Location of Revisions in Manuscript:**

- **Section 5.4.1** (newly added); (Page 17)
- References to Table 4 and Table 6 adjusted accordingly

We appreciate the reviewer’s suggestion, which has led to a more thorough and transparent explanation of our experimental findings.

**Response to Reviewer #1 - Comment 4**

**Reviewer#1 Comment 4**

*Section 5.4 compares Euclidean and hyperbolic space performance but offers a superficial analysis, neglecting to explain why hyperbolic space excels (e.g., its geometric advantages for hierarchical modeling). The comparison of hyperbolic models (Poincaré Ball vs. Lorentz) is also underdeveloped.*

**Author Response:**

We thank the reviewer for this important comment. We agree that our initial explanation in Section 5.4 lacked sufficient depth regarding the geometric rationale behind hyperbolic space and the distinct behavior of the Poincaré and Lorentz models.

To address this, we have expanded **Section 5.4.3** with the following improvements:

1. **Geometric Justification**

We provide a more detailed explanation of why hyperbolic space excels in modeling hierarchical or scale-free structures due to its exponential growth and lower distortion in representing tree-like graphs.

2. **Task-Specific Comparison: Poincaré vs. Lorentz**

We explain that the **Poincaré Ball** tends to be more effective in supervised classification tasks, benefiting from its local geometry and tighter gradients. On the other hand, the **Lorentz model** is more effective in unsupervised tasks (e.g., link prediction), where stable global distances play a larger role.

3. **General Insight**

This discussion emphasizes that the choice of hyperbolic model should consider both the graph’s structure and the training task (supervised vs. unsupervised), and reinforces the rationale behind the hybrid architecture in MOHGCAA.

**Location of Revisions in Manuscript:**

- New analysis paragraph added at the end of subsection(newly added); (Page 19)

We appreciate the reviewer’s comment, which helped us significantly improve the clarity and theoretical grounding of our model comparisons.

**Response to Reviewer #1 – Comment 5**

**Reviewer#1 Comment 5**

*For instance, the abstract’s phrase “integrating multi-order hyperbolic graph convolution with aggregated attention” could be streamlined to “integrating multi-order hyperbolic graph convolution and aggregated attention.” Section 4.1.1 redundantly repeats “and second.”*

**Author Response:**

We thank the reviewer for this helpful observation regarding language clarity and conciseness.

1. **Abstract Revision**

In response to the reviewer’s suggestion, we have revised the relevant phrase in the abstract as follows:

- **Original**:

*“…integrating multi-order hyperbolic graph convolution with aggregated attention…”*

- **Revised**:

*“…integrating multi-order hyperbolic graph convolution and aggregated attention…”*

This revision removes the unnecessary “with” construction and more clearly expresses the integrated nature of the two components, as recommended.

In addition, we conducted a full rewrite of the abstract to further improve conciseness and eliminate redundancy. The revised abstract now provides a more focused summary of our motivation, methodological novelty, and empirical contributions, while preserving technical precision and academic tone.

2. **Section 4.1.1 Redundancy Correction**

The repetitive phrase “and second” in Section 4.1.1 has been removed. The sentence structure has been revised for better flow and clarity. The updated wording now reads:

*“First, node features are projected from Euclidean space into hyperbolic space and then mapped onto the tangent space at the origin. Second, multi-order graph convolutions are applied in the tangent space… Then, the resulting features are aggregated using an attention mechanism…”*

3. **Global Improvements**

Following the reviewer’s comment, we also reviewed the manuscript for other occurrences of unnecessary repetition and verbose phrasing. Several expressions throughout the text were refined to improve overall readability and maintain a consistent academic style.

We appreciate the reviewer’s attention to language precision, which helped us significantly improve the clarity and readability of the manuscript.

We sincerely thank the editor and reviewers for their thorough and constructive feedback. Their insightful comments have substantially improved the clarity, rigor, and presentation of our manuscript. We have carefully addressed all points raised and revised the manuscript accordingly. We hope that the current version meets the standards of PLOS ONE and look forward to your further evaluation.
