# Supplementary material for: Multi-order hyperbolic graph convolution and aggregated attention for social event detection
Source: PLoS One. 2025 Dec 9;20(12):e0337540. doi: 10.1371/journal.pone.0337540 (PMC12688158; doi:10.1371/journal.pone.0337540)
Supplement: Attachment — Submitted filename: Response to Reviewer_2.docx [file pone.0337540.s003.docx]

**Dear Editor and Reviewers,**

We would like to sincerely thank you for handling our manuscript entitled

**“Multi-Order Hyperbolic Graph Convolution and Aggregated Attention for Social Event Detection”** (**Manuscript ID: PONE-D-25-09212R1**) and for the valuable comments provided by the reviewers.

We have carefully revised the manuscript according to all suggestions.

Below, we provide a detailed point-by-point response to each reviewer’s comment.

All changes have been highlighted in the revised manuscript with track changes.

**Response to Reviewer #2**

We sincerely appreciate Reviewer #2 for the positive evaluation of our revised manuscript and for recognizing the improvements made in response to the previous review round. We are pleased that the reviewer considers our revisions satisfactory and the manuscript suitable for publication. We have once again carefully proofread the entire paper to ensure linguistic accuracy, consistency in formatting, and clarity of presentation. We are grateful for the reviewer’s constructive feedback, which helped us further strengthen the quality and readability of this work.

**Response to Reviewer #3**

We sincerely thank Reviewer #3 for the detailed and constructive feedback, which has been extremely helpful in improving the clarity, rigor, and overall presentation of our work.

In response to the reviewer’s comments, we have carefully revised the manuscript, improving paragraph structure, readability, and methodological transparency.

Below, we provide a point-by-point response, with each reviewer comment quoted in italics, followed by our detailed response.

All corresponding modifications have been highlighted in the revised manuscript with track changes.

### **Comment 1: Paragraph structure and readability**

*In several cases, the text lacks structure. The paragraphs are misused. Ideally, a paragraph should contain one or at most two meanings. The authors have to split their long paragraphs to enhance readership.*

**Response:**

We appreciate this valuable comment. Following your advice, we have thoroughly revised the manuscript to improve paragraph structure and overall readability.

Long paragraphs in the *Introduction*, *Related Work*, *Methodology*, and *Experiments* sections were divided into smaller, semantically coherent units.

Each paragraph now centers on a single concept, supported by clear transitions that enhance logical flow.

These revisions significantly improve readability and align the manuscript with standard academic writing conventions.

### **Comment 2: Excessive reference chains**

*It is advised not to use chains of references (e.g., (1; 2; 3; 4; 5; 6; 7; 8)). In contrast, please use sequences of at most three references describing what each reference contributes to the article’s content.*

**Response:**

We fully agree with this comment. All chained reference groups have been revised to limit the number of consecutive citations to three, and each selected reference is now contextually explained.

For instance, in the *Introduction* section, we now describe the specific contribution of each cited work (e.g., \cite{peng2019a,zhou2020a,afyouni2022a} see p. 1).

This adjustment improves both readability and scholarly rigor by clarifying the role of each referenced study.

**Response to Reviewer #3 – Comment 3: Grammar and syntax**

*There are several syntactical and grammatical errors that have to be fixed. The authors have to carefully proofread their study.*

**Response:**

We appreciate this observation. We have thoroughly proofread the entire manuscript for grammar, syntax, punctuation, and stylistic consistency.

We have also refined several sentences for clarity and conciseness.

These revisions ensure that the manuscript adheres to standard academic English and improves overall presentation quality.

**Comment 4: Section title correction**

*Section 2 title: “Relate Work” → “Related Work”.*

**Response:**

We thank the reviewer for pointing this out. The section title has been corrected to “**Related Work**” accordingly (see Section 2, see p. 2).

**Comment 5: Lack of transition text between sections**

*It is generally advised not to stick together two section/subsection headers. Please write some introductory text between Section 2 and Subsection 2.1 headers (similarly, between 5.4 and 5.4.1).*

**Response:**

Thank you for this helpful suggestion. We have added short introductory bridging sentences between major sections and their first subsections (e.g., between Section 4.1 and 4.1.1, and between Section 5.4 and 5.4.1).

These additions improve logical continuity and make the structure easier to follow for readers.

### **Comment 6: Statistical Significance Analysis**

The statistical significance of results has not been examined. It is advised that the authors apply one or two post hoc significance analysis tests.

**Response:**

We sincerely appreciate this valuable comment. In the revised manuscript, we have added a new subsection entitled **“Statistical Stability and Significance Analysis”** (see Section 5.4.3), which clarifies the statistical reliability of our experimental results.

Specifically, all experiments were repeated three times with different random seeds, and the reported values represent the averaged performance with variance less than **0.5%** across runs.

This indicates that the model’s convergence and performance are stable.

While a full post hoc analysis (e.g., paired *t*-tests or Wilcoxon tests) was not included due to computational constraints, preliminary repeated trials confirmed that **MOHGCAA consistently outperforms the strongest baselines (HSED and HGCN)** with stable and meaningful differences.

We have also stated our plan to include full significance testing in future work.

These additions enhance the statistical transparency and reliability of our findings.

### **Comment 7: Limitation discussion**

*The authors do not describe the limitations of the proposed model. Is this approach applicable to large-scale datasets? Does it generalize well? Under which circumstances can the model fail?*

**Response:**

We thank the reviewer for highlighting this point. A new **“Limitation and Future Work”** subsection has been added at the end of the *Conclusion* section.

We acknowledge that while our framework effectively captures hierarchical dependencies, its computational cost may increase with extremely large-scale datasets due to curvature-aware attention operations.

We have discussed possible future directions, including improving efficiency through non-tangent-space approximations and exploring representations beyond tangent manifolds.

This addition enhances transparency and clarifies the scope of applicability of our proposed method.
