# Supplementary material for: Multi-order hyperbolic graph convolution and aggregated attention for social event detection
Source: PLoS One. 2025 Dec 9;20(12):e0337540. doi: 10.1371/journal.pone.0337540 (PMC12688158; doi:10.1371/journal.pone.0337540)
Supplement: Peer review history

# Peer review: Decision Letter 0

**DOI:** 10.1371/journal.pone.0337540.r001

**Authors:** Arne Johannssen

29 Jun 2025

Dear Dr. Tan,

Thank you for submitting your manuscript to PLOS ONE. After careful consideration, we feel that it has merit but does not fully meet PLOS ONE’s publication criteria as it currently stands. Therefore, we invite you to submit a revised version of the manuscript that addresses the points raised during the review process.

**Please carefully address the points raised by the reviewers.**

Please submit your revised manuscript by Aug 13 2025 11:59PM. If you will need more time than this to complete your revisions, please reply to this message or contact the journal office at plosone@plos.org . When you're ready to submit your revision, log on to https://www.editorialmanager.com/pone/ and select the 'Submissions Needing Revision' folder to locate your manuscript file.

- A rebuttal letter that responds to each point raised by the academic editor and reviewer(s). You should upload this letter as a separate file labeled 'Response to Reviewers'.
- A marked-up copy of your manuscript that highlights changes made to the original version. You should upload this as a separate file labeled 'Revised Manuscript with Track Changes'.
- An unmarked version of your revised paper without tracked changes. You should upload this as a separate file labeled 'Manuscript'.

If you would like to make changes to your financial disclosure, please include your updated statement in your cover letter. Guidelines for resubmitting your figure files are available below the reviewer comments at the end of this letter.

If applicable, we recommend that you deposit your laboratory protocols in protocols.io to enhance the reproducibility of your results. Protocols.io assigns your protocol its own identifier (DOI) so that it can be cited independently in the future. For instructions see: https://journals.plos.org/plosone/s/submission-guidelines#loc-laboratory-protocols . Additionally, PLOS ONE offers an option for publishing peer-reviewed Lab Protocol articles, which describe protocols hosted on protocols.io. Read more information on sharing protocols at https://plos.org/protocols?utm_medium=editorial-email&utm_source=authorletters&utm_campaign=protocols .

We look forward to receiving your revised manuscript.

Kind regards,

Arne Johannssen

Academic Editor

PLOS ONE

Journal Requirements:

When submitting your revision, we need you to address these additional requirements.

1. Please ensure that your manuscript meets PLOS ONE's style requirements, including those for file naming. The PLOS ONE style templates can be found at

https://journals.plos.org/plosone/s/file?id=wjVg/PLOSOne_formatting_sample_main_body.pdf and

https://journals.plos.org/plosone/s/file?id=ba62/PLOSOne_formatting_sample_title_authors_affiliations.pdf

2. Please note that PLOS ONE has specific guidelines on code sharing for submissions in which author-generated code underpins the findings in the manuscript. In these cases, we expect all author-generated code to be made available without restrictions upon publication of the work. Please review our guidelines at https://journals.plos.org/plosone/s/materials-and-software-sharing#loc-sharing-code and ensure that your code is shared in a way that follows best practice and facilitates reproducibility and reuse.

3. In your Methods section, please include additional information about your dataset and ensure that you have included a statement specifying whether the collection and analysis method complied with the terms and conditions for the source of the data.

4. We note that your Data Availability Statement is currently as follows: All relevant data are within the manuscript and its Supporting Information files.

Please confirm at this time whether or not your submission contains all raw data required to replicate the results of your study. Authors must share the “minimal data set” for their submission. PLOS defines the minimal data set to consist of the data required to replicate all study findings reported in the article, as well as related metadata and methods (https://journals.plos.org/plosone/s/data-availability#loc-minimal-data-set-definition).

For example, authors should submit the following data:

- The values behind the means, standard deviations and other measures reported;

- The values used to build graphs;

- The points extracted from images for analysis.

Authors do not need to submit their entire data set if only a portion of the data was used in the reported study.

If your submission does not contain these data, please either upload them as Supporting Information files or deposit them to a stable, public repository and provide us with the relevant URLs, DOIs, or accession numbers. For a list of recommended repositories, please see https://journals.plos.org/plosone/s/recommended-repositories.

If there are ethical or legal restrictions on sharing a de-identified data set, please explain them in detail (e.g., data contain potentially sensitive information, data are owned by a third-party organization, etc.) and who has imposed them (e.g., an ethics committee). Please also provide contact information for a data access committee, ethics committee, or other institutional body to which data requests may be sent. If data are owned by a third party, please indicate how others may request data access.

5. When completing the data availability statement of the submission form, you indicated that you will make your data available on acceptance. We strongly recommend all authors decide on a data sharing plan before acceptance, as the process can be lengthy and hold up publication timelines. Please note that, though access restrictions are acceptable now, your entire data will need to be made freely accessible if your manuscript is accepted for publication. This policy applies to all data except where public deposition would breach compliance with the protocol approved by your research ethics board. If you are unable to adhere to our open data policy, please kindly revise your statement to explain your reasoning and we will seek the editor's input on an exemption. Please be assured that, once you have provided your new statement, the assessment of your exemption will not hold up the peer review process.

[Note: HTML markup is below. Please do not edit.]

Reviewers' comments:

Reviewer's Responses to Questions

**Comments to the Author**

1. Is the manuscript technically sound, and do the data support the conclusions?

Reviewer #1: Partly

Reviewer #2: Yes

**********

2. Has the statistical analysis been performed appropriately and rigorously?

Reviewer #1: N/A

Reviewer #2: I Don't Know

**********

3. Have the authors made all data underlying the findings in their manuscript fully available?

The PLOS Data policy

Reviewer #1: Yes

Reviewer #2: Yes

**********

4. Is the manuscript presented in an intelligible fashion and written in standard English?

Reviewer #1: Yes

Reviewer #2: Yes

**********

Reviewer #1: Thank you for submitting your manuscript titled "MULTI-ORDER HYPERBOLIC GRAPH CONVOLUTION AND Aggregated Attention for Social Event Detection." After a thorough review, I recognize that your work introduces a novel framework, MOHGCAA (Multi-Order Hyperbolic Graph Convolution with Aggregated Attention), aimed at tackling the challenges of modeling hierarchical and multi-order relationships in Social Event Detection (SED). This study demonstrates potential for innovation. However, significant revisions are required due to shortcomings in originality, technical quality, experimental design, and writing quality. I recommend a Major Revision and provide detailed evaluations and suggestions below:

1. The manuscript lacks a systematic comparison with existing methods to substantiate its claimed innovations. For instance, in Section 2 ("Related Work"), methods such as MOGANED and HSED are referenced, but the unique advantages of MOHGCAA—such as specific advancements in multi-order modeling or the benefits of the attention mechanism—are not clearly delineated.

2. Critical technical details are inadequately explained. For example, Section 4.1.3 mentions using Ollivier Ricci curvature for attention weight computation but lacks specific formulas or derivations, hindering comprehension.

3. While Tables 4 and 6 show MOHGCAA’s superior performance, the analysis is shallow. For example, it does not explain why improvements are notable on mini-Twitter but modest on Cora.

4. Section 5.4 compares Euclidean and hyperbolic space performance but offers a superficial analysis, neglecting to explain why hyperbolic space excels (e.g., its geometric advantages for hierarchical modeling). The comparison of hyperbolic models (Poincaré Ball vs. Lorentz) is also underdeveloped.

5. For instance, the abstract’s phrase “integrating multi-order hyperbolic graph convolution with aggregated attention” could be streamlined to “integrating multi-order hyperbolic graph convolution and aggregated attention.” Section 4.1.1 redundantly repeats “and second.”

Reviewer #2: Authors address the challenges of social event detection by proposing a novel framework, Multi-Order

Hyperbolic Graph Convolution with Aggregated Attention. The overall research objective is clear. The conclusion of the research discusses the corresponding value provided for enterprises and public services, as well as future research plans. This part can be more specific by adding clearer application cases and scenarios. Overall, this study is suitable for publication

**********

what does this mean? ). If published, this will include your full peer review and any attached files.

If you choose “no”, your identity will remain anonymous but your review may still be made public.

**Do you want your identity to be public for this peer review?** For information about this choice, including consent withdrawal, please see our Privacy Policy

Reviewer #1: No

Reviewer #2: No

**********

[NOTE: If reviewer comments were submitted as an attachment file, they will be attached to this email and accessible via the submission site. Please log into your account, locate the manuscript record, and check for the action link "View Attachments". If this link does not appear, there are no attachment files.]

While revising your submission, please upload your figure files to the Preflight Analysis and Conversion Engine (PACE) digital diagnostic tool, https://pacev2.apexcovantage.com/ . PACE helps ensure that figures meet PLOS requirements. To use PACE, you must first register as a user. Registration is free. Then, login and navigate to the UPLOAD tab, where you will find detailed instructions on how to use the tool. If you encounter any issues or have any questions when using PACE, please email PLOS at figures@plos.org . Please note that Supporting Information files do not need this step.

# Author comment: Author response to Decision Letter 1

**DOI:** 10.1371/journal.pone.0337540.r002

15 Jul 2025

We sincerely thank the editor and reviewers for their insightful and constructive comments.

In response, we have carefully revised the manuscript and prepared a detailed point-by-point “Response to Reviewers” document, which has been uploaded as a separate file.

All major and minor concerns have been addressed. We have also provided a clean version of the revised manuscript and a tracked-changes version as required.

We respectfully submit the revised manuscript for your further consideration.

Attachment Submitted filename: Response to Reviewer.docx

# Peer review: Decision Letter 1

**DOI:** 10.1371/journal.pone.0337540.r003

**Authors:** Arne Johannssen

20 Oct 2025

Dear Dr. Tan,

Thank you for submitting your manuscript to PLOS ONE. After careful consideration, we feel that it has merit but does not fully meet PLOS ONE’s publication criteria as it currently stands. Therefore, we invite you to submit a revised version of the manuscript that addresses the points raised during the review process.

**Please carefully address the concerns raised by the reviewer.**

Please submit your revised manuscript by Dec 04 2025 11:59PM. If you will need more time than this to complete your revisions, please reply to this message or contact the journal office at plosone@plos.org . When you're ready to submit your revision, log on to https://www.editorialmanager.com/pone/ and select the 'Submissions Needing Revision' folder to locate your manuscript file.

- A rebuttal letter that responds to each point raised by the academic editor and reviewer(s). You should upload this letter as a separate file labeled 'Response to Reviewers'.
- A marked-up copy of your manuscript that highlights changes made to the original version. You should upload this as a separate file labeled 'Revised Manuscript with Track Changes'.
- An unmarked version of your revised paper without tracked changes. You should upload this as a separate file labeled 'Manuscript'.

If you would like to make changes to your financial disclosure, please include your updated statement in your cover letter. Guidelines for resubmitting your figure files are available below the reviewer comments at the end of this letter.

If applicable, we recommend that you deposit your laboratory protocols in protocols.io to enhance the reproducibility of your results. Protocols.io assigns your protocol its own identifier (DOI) so that it can be cited independently in the future. For instructions see: https://journals.plos.org/plosone/s/submission-guidelines#loc-laboratory-protocols . Additionally, PLOS ONE offers an option for publishing peer-reviewed Lab Protocol articles, which describe protocols hosted on protocols.io. Read more information on sharing protocols at https://plos.org/protocols?utm_medium=editorial-email&utm_source=authorletters&utm_campaign=protocols .

We look forward to receiving your revised manuscript.

Kind regards,

Arne Johannssen

Academic Editor

PLOS ONE

Journal Requirements:

If the reviewer comments include a recommendation to cite specific previously published works, please review and evaluate these publications to determine whether they are relevant and should be cited. There is no requirement to cite these works unless the editor has indicated otherwise. 

[Note: HTML markup is below. Please do not edit.]

Reviewers' comments:

Reviewer's Responses to Questions

**Comments to the Author**

Reviewer #2: All comments have been addressed

Reviewer #3: (No Response)

**********

2. Is the manuscript technically sound, and do the data support the conclusions?

Reviewer #2: Yes

Reviewer #3: Yes

**********

3. Has the statistical analysis been performed appropriately and rigorously?

Reviewer #2: Yes

Reviewer #3: N/A

**********

4. Have the authors made all data underlying the findings in their manuscript fully available?

The PLOS Data policy

Reviewer #2: Yes

Reviewer #3: Yes

**********

5. Is the manuscript presented in an intelligible fashion and written in standard English?

Reviewer #2: Yes

Reviewer #3: Yes

**********

Reviewer #2: The author answered the reviewer's questions and made necessary modifications to some of the content. The adjusted research has answered previous doubts and is suitable for publication.

Reviewer #3: This paper introduces a new framework, called Multi-Order Hyperbolic Graph Convolution with Aggregated Attention (MOHGCAA), with the aim of enhancing the performance of social event detection.

The paper’s quality is good and the contribution seems to be significant. The transition from Euclidean spaces to hyperbolic spaces for social event detection is an interesting alternative in my opinion.

1. In several cases, the text lacks structure. The paragraphs are misused. Ideally, a paragraph should contain one or at most two meanings. The authors have to split their long paragraphs to enhance readership.

2. It is advised not to use chains of references (e.g. (1; 2; 3; 4; 5; 6; 7; 8)). In contrast, please use sequences of at most three references describing what each reference contributes to the article’s content.

3. There are several syntactical and grammatical errors that have to be fixed. The authors have to carefully proofread their study.

4. Section 2 title: “Relate Work” -> “Related Work”

5. It is generally advised not to stick together two section/subsection headers. Please write some introductory text between Section 2 and Subsection 2.1 headers (similarly, between 5.4 and 5.4.1).

6. The statistical significance of results has not been examined. It is advised that the authors apply one or two post hoc significance analysis tests.

7. The authors do not describe the limitations of the proposed model. Is this approach applicable to large-scale datasets? Does it generalize well? Under which circumstances can the model fail?

**********

what does this mean? ). If published, this will include your full peer review and any attached files.

If you choose “no”, your identity will remain anonymous but your review may still be made public.

**Do you want your identity to be public for this peer review?** For information about this choice, including consent withdrawal, please see our Privacy Policy

Reviewer #2: No

Reviewer #3: No

**********

[NOTE: If reviewer comments were submitted as an attachment file, they will be attached to this email and accessible via the submission site. Please log into your account, locate the manuscript record, and check for the action link "View Attachments". If this link does not appear, there are no attachment files.]

To ensure your figures meet our technical requirements, please review our figure guidelines: https://journals.plos.org/plosone/s/figures 

You may also use PLOS’s free figure tool, NAAS, to help you prepare publication quality figures: https://journals.plos.org/plosone/s/figures#loc-tools-for-figure-preparation. 

NAAS will assess whether your figures meet our technical requirements by comparing each figure against our figure specifications.

# Author comment: Author response to Decision Letter 2

**DOI:** 10.1371/journal.pone.0337540.r004

27 Oct 2025

Dear Reviewers,

We sincerely thank you for your constructive and detailed feedback. Following your guidance, we have thoroughly revised the manuscript to address all comments raised by Reviewer #3 and the editorial office. The main revisions include:

1. Paragraph restructuring and readability: Long paragraphs in the Introduction, Related Work, and Methodology sections have been reorganized into shorter, semantically coherent units to enhance readability.

2. Reference revision: All excessive citation chains have been reduced to a maximum of three references, with explicit contextual explanation of each cited work.

3. Language polishing: The entire manuscript was carefully proofread to correct grammatical and syntactic issues and ensure academic clarity.

4. Section title correction: The title “Relate Work” has been corrected to “Related Work.”

5. Improved transitions: Introductory sentences have been added between consecutive section/subsection headers (e.g., Sections 4–4.1 and 5.4–5.4.1) to improve logical flow.

6. Statistical reliability: A new subsection, Statistical Stability and Significance Analysis, has been added to discuss experimental reproducibility and the statistical robustness of our results.

7. Model limitations: A Limitation and Future Work subsection has been added to the Conclusion, discussing scalability, potential failure cases, and future directions.

All changes have been marked in the revised manuscript with track changes. We believe these revisions have substantially improved the clarity, rigor, and presentation of the paper, and we hope it now meets the publication standards of PLOS ONE.

Sincerely,

Yao Liu (on behalf of all authors)

Department of Management and Media, Chengdu University of Technology

School of Computer Sciences, Universiti Sains Malaysia

Attachment Submitted filename: Response to Reviewer_2.docx

# Peer review: Decision Letter 2

**DOI:** 10.1371/journal.pone.0337540.r005

**Authors:** Arne Johannssen

10 Nov 2025

Multi-Order Hyperbolic Graph Convolution and Aggregated Attention for Social Event Detection

PONE-D-25-09212R2

Dear Dr. Tan,

We’re pleased to inform you that your manuscript has been judged scientifically suitable for publication and will be formally accepted for publication once it meets all outstanding technical requirements.

Within one week, you’ll receive an e-mail detailing the required amendments. When these have been addressed, you’ll receive a formal acceptance letter and your manuscript will be scheduled for publication.

An invoice will be generated when your article is formally accepted. Please note, if your institution has a publishing partnership with PLOS and your article meets the relevant criteria, all or part of your publication costs will be covered. Please make sure your user information is up-to-date by logging into Editorial Manager at Editorial Manager®  and clicking the ‘Update My Information' link at the top of the page. For questions related to billing, please contact billing support .

If your institution or institutions have a press office, please notify them about your upcoming paper to help maximize its impact. If they’ll be preparing press materials, please inform our press team as soon as possible -- no later than 48 hours after receiving the formal acceptance. Your manuscript will remain under strict press embargo until 2 pm Eastern Time on the date of publication. For more information, please contact onepress@plos.org.

Kind regards,

Arne Johannssen

Academic Editor

PLOS ONE

Additional Editor Comments (optional):

Reviewers' comments:

Reviewer's Responses to Questions

**Comments to the Author**

Reviewer #2: (No Response)

Reviewer #3: All comments have been addressed

**********

2. Is the manuscript technically sound, and do the data support the conclusions?

Reviewer #2: Yes

Reviewer #3: Yes

**********

3. Has the statistical analysis been performed appropriately and rigorously?

Reviewer #2: Yes

Reviewer #3: N/A

**********

4. Have the authors made all data underlying the findings in their manuscript fully available?

The PLOS Data policy

Reviewer #2: Yes

Reviewer #3: Yes

**********

5. Is the manuscript presented in an intelligible fashion and written in standard English?

Reviewer #2: Yes

Reviewer #3: Yes

**********

Reviewer #2: After modification, the author not only answered some of the previous doubts, but also made the experiment more rigorous. The added content in the conclusion section also leaves possibilities for future related research. Therefore, the article is suitable for publication

Reviewer #3: (No Response)

**********

what does this mean? ). If published, this will include your full peer review and any attached files.

If you choose “no”, your identity will remain anonymous but your review may still be made public.

**Do you want your identity to be public for this peer review?** For information about this choice, including consent withdrawal, please see our Privacy Policy

Reviewer #2: No

Reviewer #3: No

**********

# Editor report: Acceptance letter

**DOI:** 10.1371/journal.pone.0337540.r006

**Authors:** Arne Johannssen

PONE-D-25-09212R2

PLOS ONE

Dear Dr. Tan,

I'm pleased to inform you that your manuscript has been deemed suitable for publication in PLOS ONE. Congratulations! Your manuscript is now being handed over to our production team.

At this stage, our production department will prepare your paper for publication. This includes ensuring the following:

* All references, tables, and figures are properly cited

* All relevant supporting information is included in the manuscript submission,

* There are no issues that prevent the paper from being properly typeset

You will receive further instructions from the production team, including instructions on how to review your proof when it is ready. Please keep in mind that we are working through a large volume of accepted articles, so please give us a few days to review your paper and let you know the next and final steps.

Lastly, if your institution or institutions have a press office, please let them know about your upcoming paper now to help maximize its impact. If they'll be preparing press materials, please inform our press team within the next 48 hours. Your manuscript will remain under strict press embargo until 2 pm Eastern Time on the date of publication. For more information, please contact onepress@plos.org.

You will receive an invoice from PLOS for your publication fee after your manuscript has reached the completed accept phase. If you receive an email requesting payment before acceptance or for any other service, this may be a phishing scheme. Learn how to identify phishing emails and protect your accounts at https://explore.plos.org/phishing.

If we can help with anything else, please email us at customercare@plos.org.

Thank you for submitting your work to PLOS ONE and supporting open access.

Kind regards,

PLOS ONE Editorial Office Staff

on behalf of

Profesor Arne Johannssen

Academic Editor

PLOS ONE
